# Supplementary material for: Human iPSCs-based modeling unveils SETBP1 as a driver of chromatin rewiring in GATA2 deficiency
Source: Nat Commun. 2025 Nov 17;16:10035. doi: 10.1038/s41467-025-65806-9 (PMC12623428; doi:10.1038/s41467-025-65806-9)
Supplement: Supplementary file 1 — Supplementary Information [file 41467_2025_65806_MOESM1_ESM.pdf]

Title: Human iPSCs-based modeling unveils SETBP1 as a driver of chromatin rewiring in GATA2 deficiency

Authors: Joan Pera(1,2,3), Damia Romero-Moya(1,2), Eric Torralba-Sales(1,2), Rebecca Andersson(2,4), Violeta García-Hernández(5,6,†), Maria Magallon-Mosella(1,2), Maximiliano Distefano(1,2), Clara Berenguer Balaguer(7), Julio Castaño(8), Francesca De Giorgio(1,2), Zhichao Qiu(1,2,9,10), Arnau Iglesias(5,6,11), Paulina Spurk(12,13), Sara Montserrat-Vazquez(2,4), Lorenzo Pasquali(7), Zhuobin Liang(9), Albert Català(14,15), M Carolina Florian(2,4,16,17), Marcin W Wlodarski(18), Anna Bigas(5,6,11), Oskar Marin-Bejar*(1,2,19) & Alessandra Giorgetti*(1,2,16,20,21).

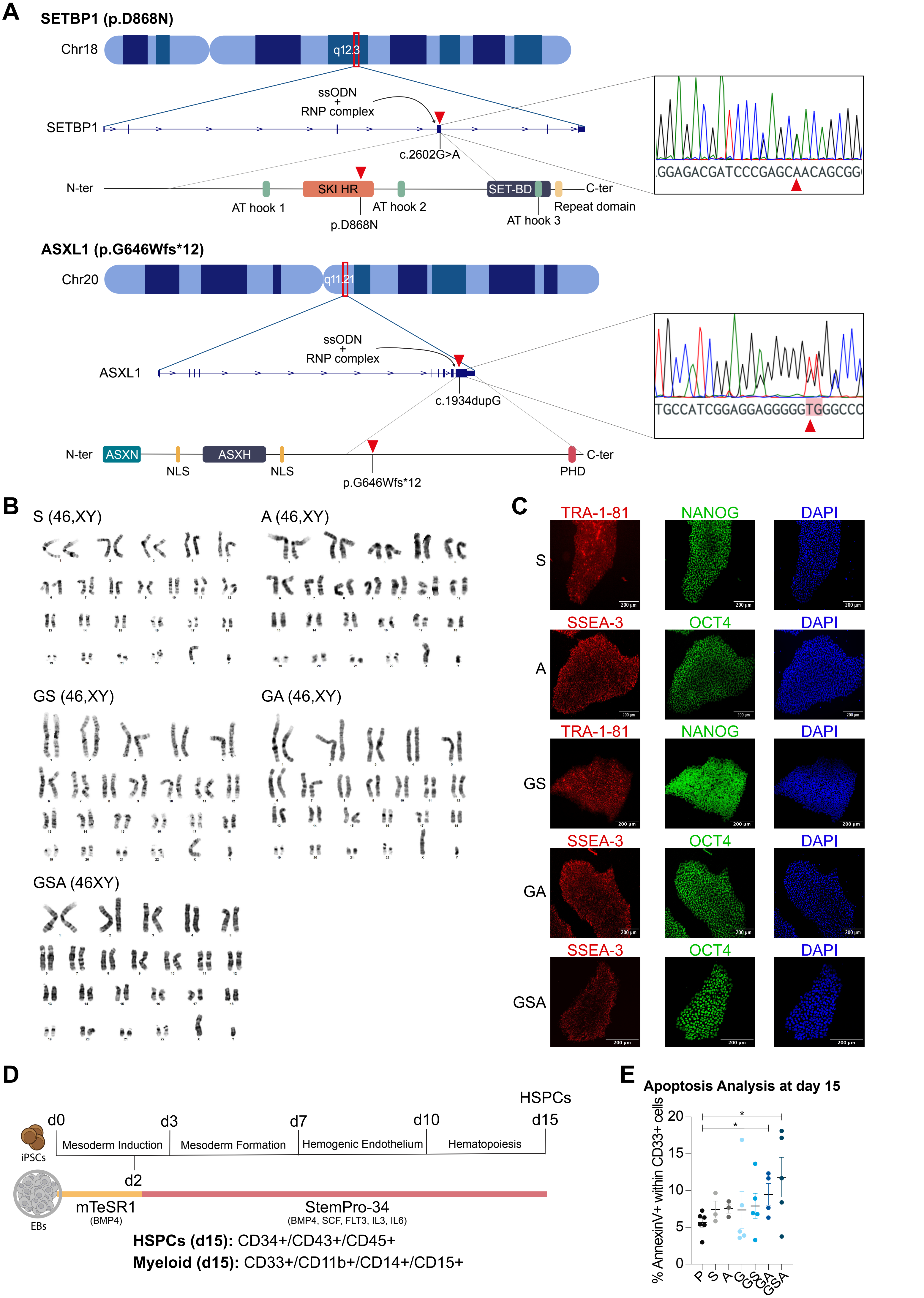

A
SETBP1 (p.D868N)
Chr18
q12.3
ssODN + RNP complex
SETBP1
c.2602G>A
N-ter
SKI HR
SET-BD
C-ter
AT hook 1
AT hook 2
AT hook 3
Repeat domain
p.D868N
GGAGACGATCCCGAGCAACAGCGG
ASXL1 (p.G646Wfs*12)
Chr20
q11.21
ssODN + RNP complex
ASXL1
c.1934dupG
TGCCATCGGAGGAGGGGGTGGGCCC
N-ter
ASXN
ASXH
NLS
NLS
p.G646Wfs*12
PHD
C-ter
B
S (46,XY)
A (46,XY)
GS (46,XY)
GA (46,XY)
GSA (46XY)
C
TRA-1-81
NANOG
DAPI
SSEA-3
OCT4
S
A
GS
GA
GSA
200 µm
D
iPSCs
EBs
d0
d2
d3
d7
d10
d15
HSPCs
Mesoderm Induction
Mesoderm Formation
Hemogenic Endothelium
Hematopoiesis
mTeSR1
(BMP4)
StemPro-34
(BMP4, SCF, FLT3, IL3, IL6)
HSPCs (d15): CD34+/CD43+/CD45+
Myeloid (d15): CD33+/CD11b+/CD14+/CD15+
E
Apoptosis Analysis at day 15
% AnnexinV+ within CD33+ cells
20
15
10
5
0
P
S
A
GS
GA
GSA
*
*

Supplementary Figure 1. iPSCs line generation and characterization.

(A) CRISPR/Cas9-mediated genome editing strategy for introducing *SETBP1* and *ASXL1* mutations. Schematic representation of the genomic loci of *SETBP1* and *ASXL1*, including the design of sgRNAs and ssODNs used to introduce heterozygous mutations c.2602G>A (*SETBP1*) and c.1934dupG (*ASXL1*). Targeted gene editing was validated by Sanger sequencing, confirming successful introduction of the desired point mutation and duplication, respectively.

(B) Representative karyotype of S, A, GS, GA, and GSA hiPSCs lines. G-banding analysis was performed to assess chromosomal integrity, confirming normal karyotypes across all lines shown.

(C) Immunofluorescence analysis of S, A, GS, GA, and GSA lines for pluripotency markers. The colonies express the embryonic markers, SSEA-3, TRA-1-81, and the transcription factors OCT4 and NANOG.

(D) Schematic diagram of hematopoietic differentiation protocol based on EB generation and phenotypic characterization at day 10 and 15 of differentiation.

(E) Apoptosis analysis of CD33+ cells at day 15 of EB differentiation. Quantification of Annexin V cells was performed by flow cytometry to assess apoptotic cell population within the CD33+ compartment at day 15 of EB differentiation. Data represent the mean ± SEM of independent biological experiments (P=6, S=3, A=3, G=5, GS=5, GA=4, and GSA=5).

P= Parental, S=SETBP1 mutant, A=ASXL1 mutant, G=GATA2, GS= GATA2-SETBP1 mutant, GA= GATA2-ASXL1 mutant, GSA = GATA2-SETBP1-ASXL1 mutant.

Statistical analysis: Data with a normal distribution were analyzed using the two-sided Student's *t*-test, while non-normally distributed data were analyzed using the two-sided Mann–Whitney test. Statistical significance was indicated as follows: $p<0.05$ (*), $p<0.001$ (**), and $p<0.0001$ (****). Exact *p*-values can be found on supplementary source data.

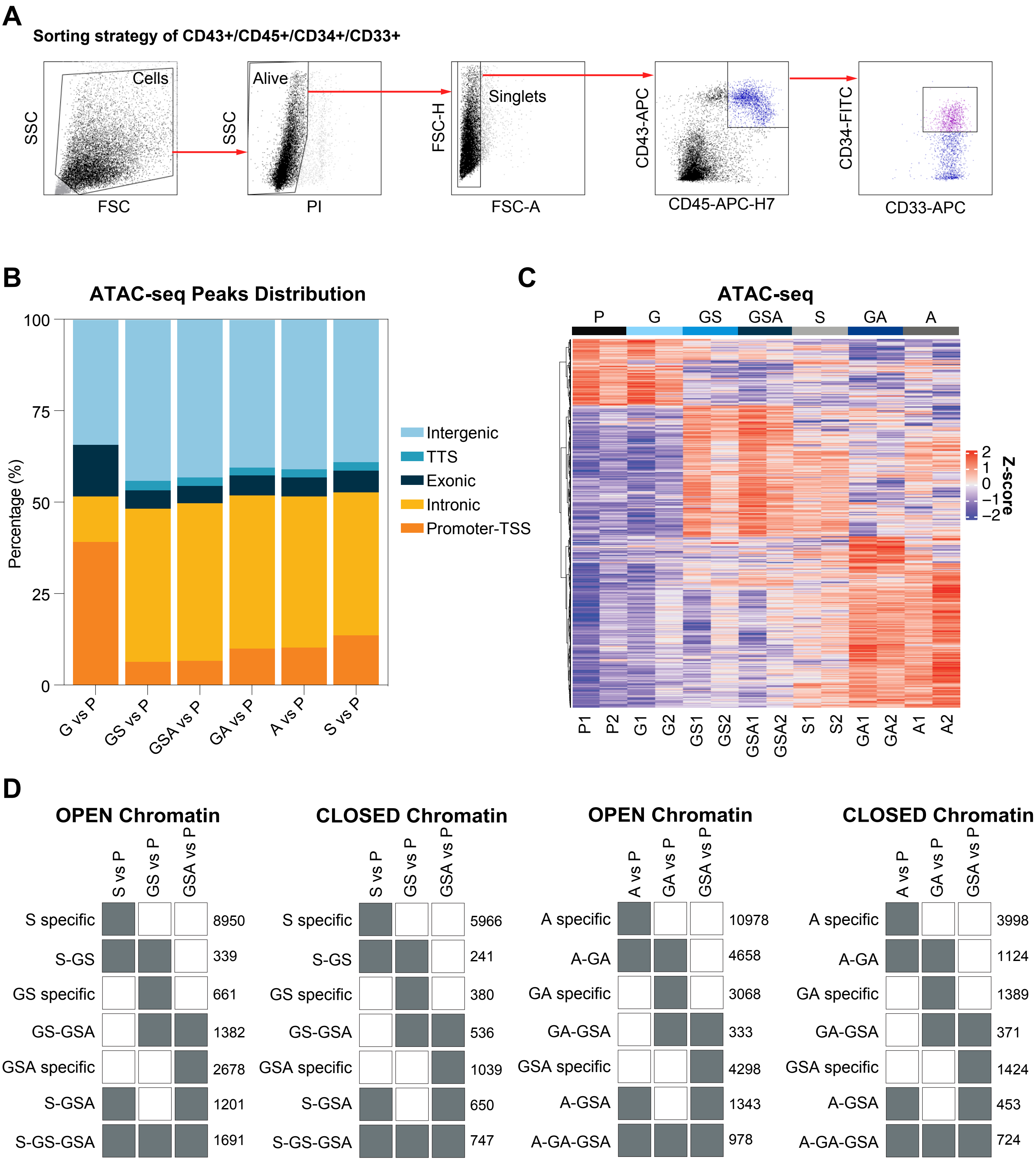
A
Sorting strategy of CD43+/CD45+/CD34+/CD33+
Cells
SSC
FSC
Alive
SSC
PI
Singlets
FSC-H
FSC-A
CD43-APC
CD45-APC-H7
CD34-FITC
CD33-APC
B
ATAC-seq Peaks Distribution
Percentage (%)
100
75
50
25
0
G vs P
GS vs P
GSA vs P
GA vs P
A vs P
S vs P
Intergenic
TTS
Exonic
Intronic
Promoter-TSS
C
ATAC-seq
P
G
GS
GSA
S
GA
A
Z-score
2
1
0
−1
−2
P1
P2
G1
G2
GS1
GS2
GSA1
GSA2
S1
S2
GA1
GA2
A1
A2
D
OPEN Chromatin
S vs P
GS vs P
GSA vs P
S specific 8950
S-GS 339
GS specific 661
GS-GSA 1382
GSA specific 2678
S-GSA 1201
S-GS-GSA 1691
CLOSED Chromatin
S vs P
GS vs P
GSA vs P
S specific 5966
S-GS 241
GS specific 380
GS-GSA 536
GSA specific 1039
S-GSA 650
S-GS-GSA 747
OPEN Chromatin
A vs P
GA vs P
GSA vs P
A specific 10978
A-GA 4658
GA specific 3068
GA-GSA 333
GSA specific 4298
A-GSA 1343
A-GA-GSA 978
CLOSED Chromatin
A vs P
GA vs P
GSA vs P
A specific 3998
A-GA 1124
GA specific 1389
GA-GSA 371
GSA specific 1424
A-GSA 453
A-GA-GSA 724

Supplementary Figure 2. Sorting strategy of EB-derived HSPCs and their ATAC-seq profile.

(A) Gating strategy for isolation of live $CD43^{+}CD45^{+}CD34^{+}CD33^{+}$ cells from differentiated embryoid bodies (EBs). Dead cells were excluded using propidium iodide (PI) as viability dye, followed by singlet discrimination using FSC-H vs FSC-A gating. Live singlets were then gated for $CD43^{+}CD45^{+}$ expression, followed by selection of $CD34^{+}CD33^{+}$ cells for sorting. Differential chromatin accessibility was assessed using edgeR and limma. Count data were modeled with a negative binomial distribution, and moderated t-tests were performed. Reported p-values are two-sided and adjusted for multiple comparisons using the Benjamini–Hochberg false discovery rate (FDR).

(B) Distribution of ATAC-seq differential accessible peaks (DAPs) across genomic regions. Bar plot representing the proportion of accessible chromatin regions mapped to intergenic (light blue), transcription termination site (TTS, teal), exonic (dark blue), intronic (yellow), and promoter–transcription start site (Promoter-TSS, orange) genomic features. Differential chromatin accessibility was assessed using edgeR and limma. Count data were modeled with a negative binomial distribution, and moderated t-tests were performed. Reported p-values are two-sided and adjusted for multiple comparisons using the Benjamini–Hochberg false discovery rate (FDR).

(C) Genomic distribution of DAPs in mutant hiPSCs compared to parental (P) line. Heatmap displays the relative enrichment of DAPs across genomic regions. Mutant conditions show distinct chromatin accessibility patterns. Data were derived from ATAC-seq, the color scale represents normalized accessibility scores. DAPs were defined by absolute log2FC>0.5 and adjusted *p*-value <0.05. Differential chromatin accessibility was assessed using edgeR and limma. Count data were modeled with a negative binomial distribution, and moderated t-tests were performed. Reported p-values are two-sided and adjusted for multiple comparisons using the Benjamini–Hochberg false discovery rate (FDR).

(D) The representation of the intersection between open and closed DAPs across indicated comparisons. The plot illustrates the overlap between more accessible and less accessible DAPs in the indicated pairwise comparisons: A vs P, GA vs P, GSA vs P, S vs P, GS vs P, and GSA vs P. Each box represents the distribution of shared DAPs across comparisons, highlighting common and distinct transcriptional responses relative to the P condition. Overlaps were quantified based on consistent directionality (more accessible or less accessible).

P=Parental, S=SETBP1 mutant, A=ASXL1 mutant, G=GATA2 mutant, GS= GATA2-SETBP1 mutant, GA= GATA2-ASXL1 mutant, GSA = GATA2-SETBP1-ASXL1 mutant.

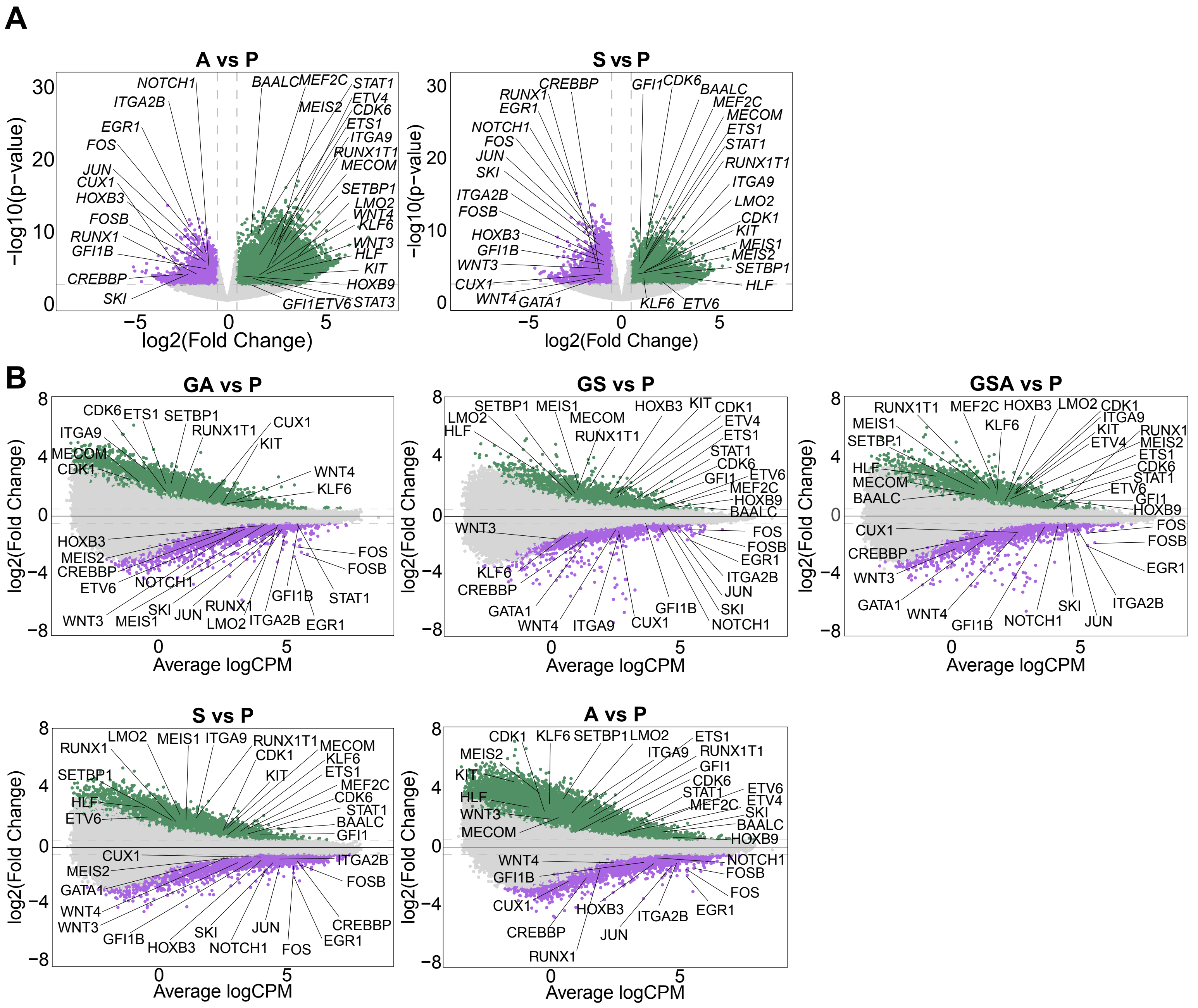

A
A vs P
S vs P
−log10(p−value)
log2(Fold Change)
NOTCH1 ITGA2B EGR1 FOS JUN CUX1 HOXB3 FOSB RUNX1 GFI1B CREBBP SKI
BAALC MEF2C STAT1 MEIS2 ETV4 CDK6 ETS1 ITGA9 RUNX1T1 MECOM SETBP1 LMO2 WNT4 KLF6 WNT3 HLF KIT HOXB9 GFI1 ETV6 STAT3
CREBBP RUNX1 EGR1 NOTCH1 FOS JUN SKI ITGA2B FOSB HOXB3 GFI1B WNT3 CUX1 WNT4 GATA1
GFI1 CDK6 BAALC MEF2C MECOM ETS1 STAT1 RUNX1T1 ITGA9 LMO2 CDK1 KIT MEIS1 MEIS2 SETBP1 HLF KLF6 ETV6
B
GA vs P
GS vs P
GSA vs P
S vs P
A vs P
log2(Fold Change)
Average logCPM

Supplementary Figure 3. Volcano and MA plots showing more accessible and less accessible peaks compared to P.

(A) Volcano plots represent differential chromatin accessibility in A vs P, and S vs P comparisons. Each point represents an individual chromatin accessible region, with the log2FC>0.5 in accessibility (x-axis) plotted against the –log10(*p*-value) (y-axis). Significant peaks defined by *p*-value<0.05 and absolute log2|FC|>0.5, are colored. Genes associated with significant DAPs are indicated. Differential chromatin accessibility was assessed using edgeR and limma. Count data were modeled with a negative binomial distribution, and moderated t-tests were performed. Reported p-values are two-sided and adjusted for multiple comparisons using the Benjamini–Hochberg false discovery rate (FDR).

(B) MvA plots showing differential chromatin accessibility in GA vs P, GS vs P, GSA vs P, A vs P, and S vs P. The x-axis indicates the average expression (mean of counts per million, in log scale), while the y-axis shows the log2 fold change between the compared conditions. Points highlighted in green indicate significant peaks (*p*-value < 0.05) more accessible in the comparison versus P, whereas points in purple represent significant peaks (*p*-value < 0.05) less accessible relative to the reference.

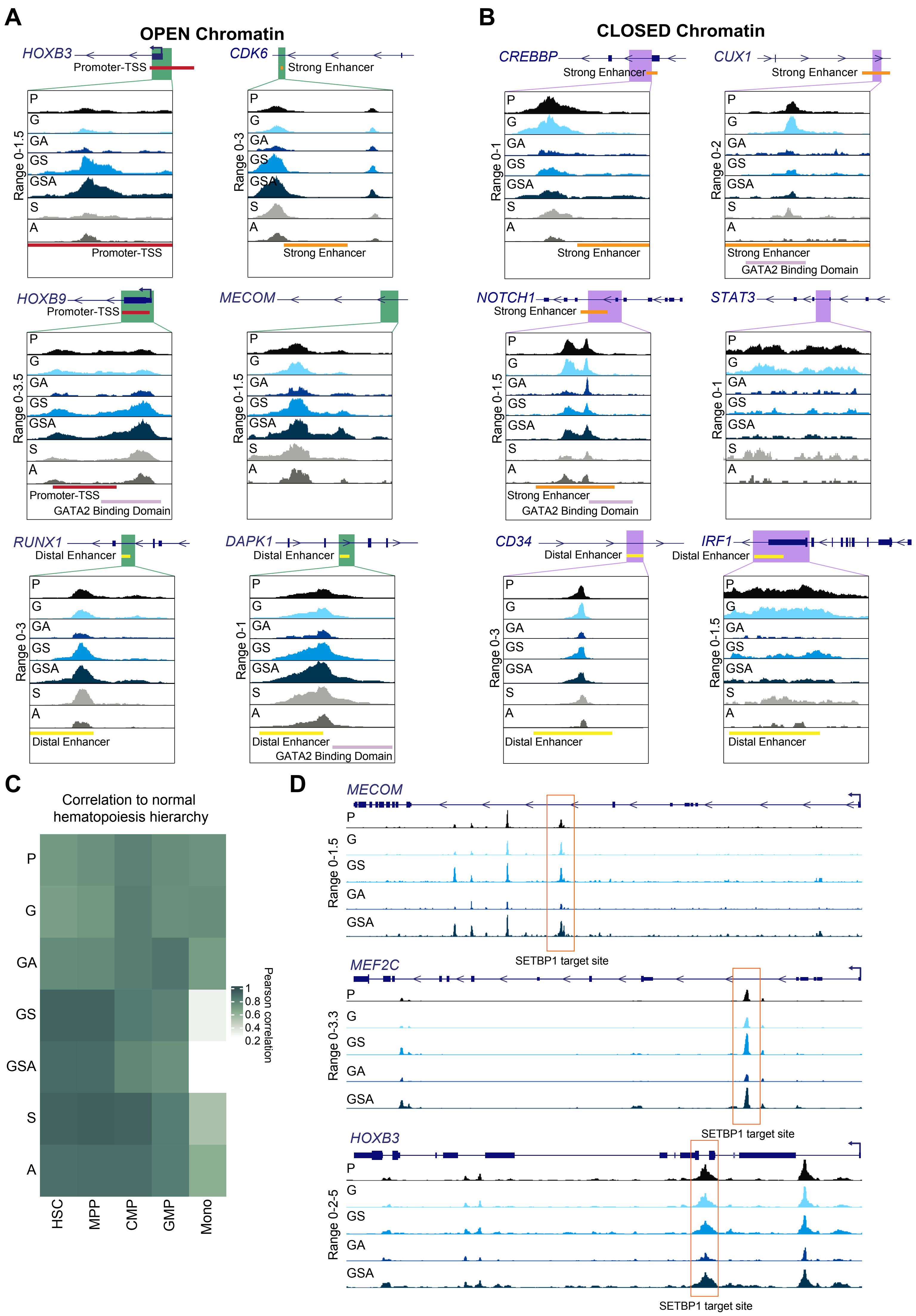
A
OPEN Chromatin
HOXB3
Promoter-TSS
CDK6
Strong Enhancer
Range 0-1.5
Range 0-3
P
G
GA
GS
GSA
S
A
HOXB9
MECOM
GATA2 Binding Domain
Range 0-3.5
RUNX1
DAPK1
Distal Enhancer
Range 0-1
B
CLOSED Chromatin
CREBBP
CUX1
Range 0-2
NOTCH1
STAT3
CD34
IRF1
C
Correlation to normal hematopoiesis hierarchy
HSC
MPP
CMP
GMP
Mono
Pearson correlation
1
0.8
0.6
0.4
0.2
D
MECOM
MEF2C
HOXB3
SETBP1 target site
Range 0-3.3
Range 0-2.5

Supplementary Figure 4. Differentially regulation of chromatin accessibility in GA, GS and GSA hiPSCs.

(A) Representative Integrative Genomics Viewer (IGV) visualization and regulatory annotations for *HOXB3*, *CDK6*, *HOXB9*, *MECOM*, *RUNX1*, and *DAPK1* loci. Tracks display chromatin accessibility profiles for parental (P, black) and mutant conditions (G, light blue; GS, blue; GA, navy blue; GSA, dark blue). Display of the genomic interval exhibiting the highest $\log_2$ fold change (logFC) from the comparison of condition GSA versus condition P. Regulatory elements are annotated as strong enhancers (orange), distal enhancers (yellow), and promoter-transcription start site (TSS) regions (red), Genehancer track (version 2) on the UCSC Genome Browser(db=hg38). The green box highlights the genomic interval shown

(B) Representative IGV visualization and regulatory annotations for *CREBBP, CUX1, NOTCH1, STAT3, CD34,* and *IRF1* loci. Tracks display chromatin accessibility profiles for parental (P, black) and mutant conditions (G, light blue; GS, blue; GA, navy blue; GSA, dark blue). Display of the genomic interval exhibiting the highest $\log_2$ fold change (logFC) from the comparison of condition GSA versus condition P. Regulatory elements are annotated as strong enhancers (orange), distal enhancers (yellow), and promoter-transcription start site (TSS) regions (red), annotated regulatory elements are indicated: strong enhancer (orange), distal enhancer (yellow) and promoter-TSS (red), Genehancer track (version 2) on the UCSC Genome Browser(db=hg38). The purple box highlights the genomic interval shown.

(C) Heatmap showing Pearson correlation values of normalized read counts for ATAC-seq differential accessible peaks (DAPs) that overlap between our clonal evolution stages (P, G, GA, GS, GSA) and primary normal hematopoietic cell subpopulations (Hematopoietic Stem Cell [HSC], Multi-Potent Progenitor [MPP], Common Myeloid Progenitor [CMP], Granulocyte-Monocyte Progenitor [GMP], and Monocytes from Corces *et al.*[58].

(D) Integration of ATAC-seq differential accessibility with SETBP1 binding profiles. Overlay of DAPs with SETBP1 ChIP-seq targets from Piazza *et al.*[59], showing significant enrichment at shared regulatory regions. Example loci (*MECOM*, *MEF2C*, and *HOXB3*) demonstrate coordinated changes in chromatin accessibility (ATAC-seq) at SETBP1-bound sites.

P=Parental, G=GATA2 mutant, GS= GATA2-SETBP1 mutant, GA= GATA2-ASXL1 mutant, GSA = GATA2-SETBP1-ASXL1 mutant.

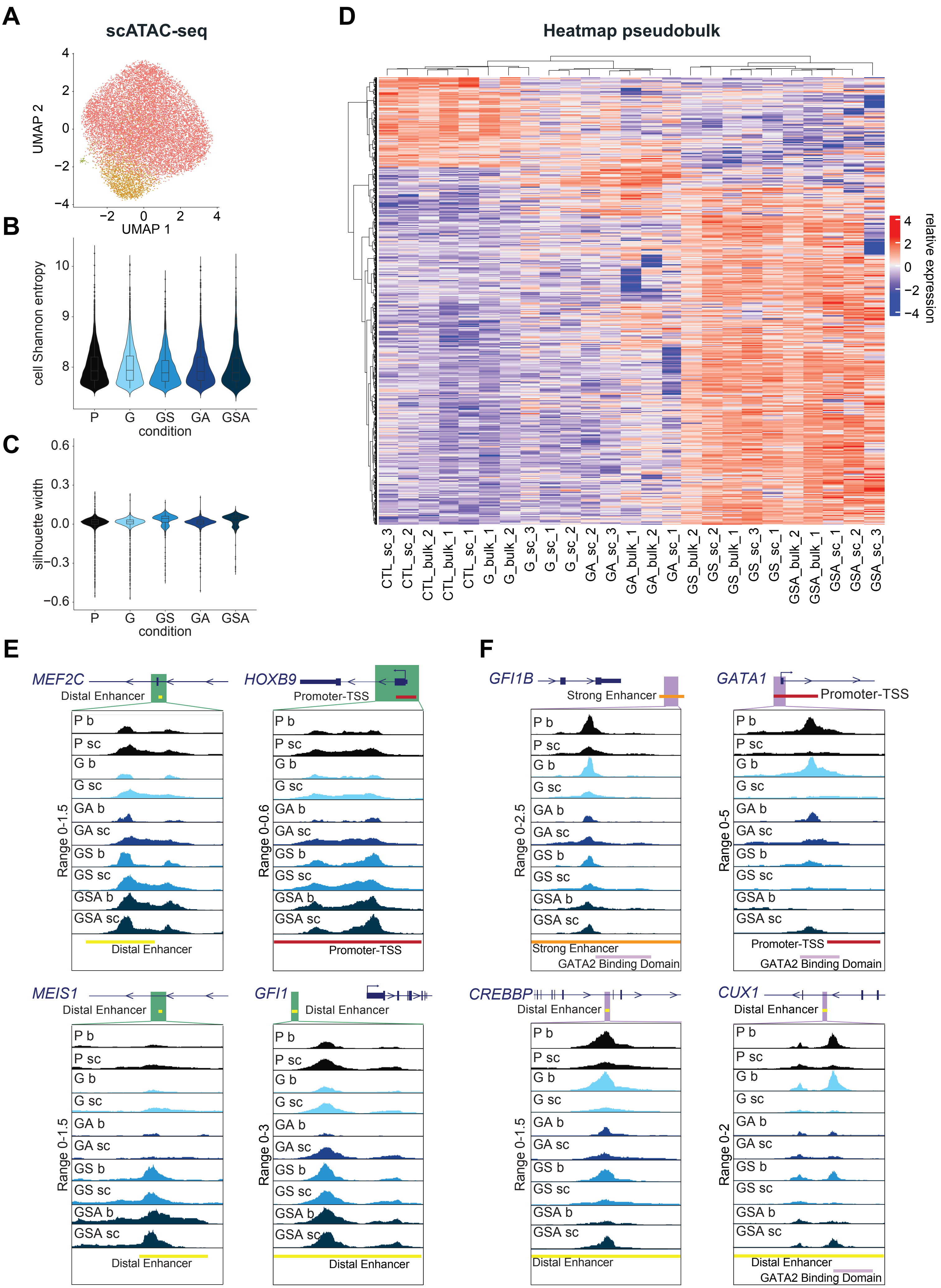
A
scATAC-seq
UMAP 1
UMAP 2
B
cell Shannon entropy
condition
P G GS GA GSA
C
silhouette width
D
Heatmap pseudobulk
relative expression
CTL_sc_3 CTL_sc_2 CTL_bulk_2 CTL_bulk_1 CTL_sc_1 G_bulk_1 G_bulk_2 G_sc_3 G_sc_1 G_sc_2 GA_sc_2 GA_sc_3 GA_bulk_1 GA_bulk_2 GA_sc_1 GS_bulk_2 GS_sc_2 GS_bulk_1 GS_sc_3 GS_sc_1 GSA_bulk_2 GSA_bulk_1 GSA_sc_1 GSA_sc_2 GSA_sc_3
E
MEF2C
Distal Enhancer
HOXB9
Promoter-TSS
MEIS1
GFI1
P b
P sc
G b
G sc
GA b
GA sc
GS b
GS sc
GSA b
GSA sc
Range 0-1.5
Range 0-0.6
Range 0-3
F
GFI1B
Strong Enhancer
GATA1
CREBBP
CUX1
GATA2 Binding Domain
Range 0-2.5
Range 0-5
Range 0-2

Supplementary Figure 5. scATAC-seq analysis and bulk integration reveals condition specific regulatory landscapes.

(A) UMAP plot display the dimensionality reduction of chromatin accessibility distribution of single cells (with 16270 cells).

(B) Cell Shannon entropy across conditions. Plot displays entropy distributions for P (black), G (light blue), GS (blue), GA (navy blue), and GSA (dark blue), allowing comparison of variability across groups.

(C) Silhouette width plot of clustering performance. The silhouette coefficient was calculated for each cell to evaluate cluster cohesion and separation. Each dot represents an individual cell, ordered by silhouette value within its assigned cluster. Values close to +1 indicate cells that are well matched to their own cluster and distinct from neighboring clusters, values near 0 suggest overlap between clusters, and negative values indicate potential misclassification.

(D) Heatmap of genomic distribution of differentially accessible peaks (DAPs) of pseudobulk- sc ATAC-seq samples and bulk ATAC-seq datasets. Color intensity represents the relative upregulation (red) or downregulation (blue) of DAPs across genomic annotations.

P=Parental, G=GATA2 mutant, GS= GATA2-SETBP1 mutant, GA= GATA2-ASXL1 mutant, GSA = GATA2-SETBP1-ASXL1 mutant.

(E) Representative ATAC-seq genome tracks illustrating open differentially accessible peaks (DAPs), defined by adjusted pvalue<0.05 and absolute log2|FC|>0.5. Representative genome browser tracks for *MEF2C, HOXB9, MEIS1,* and *GFI1* loci are shown. ATAC-seq signal is color-coded by condition: P (black), G (light blue), GS (blue), GA (navy blue), GSA (dark blue). Tracks for ATAC-seq bulk data are differentiated with a letter b after name condition (i.e. P b). Tracks for pseudobulk-scATAC-seq are labeled with "sc" after name condition (i.e. P sc). Annotated regulatory elements are indicated: strong enhancer (orange), distal enhancer (yellow) and promoter-TSS (red), Genehancer track (version 2) on the UCSC Genome Browser(db=hg38). The green box marks the genomic interval displayed for each gene locus.

(F) Representative genome browser tracks for the *GFI1B, GATA1, CREBBP,* and *CUX1* loci are shown. ATAC-seq signal is color-coded by condition: P (black), G (light blue), GS (blue), GA (navy blue), GSA (dark blue), Tracks for ATAC-seq bulk data are differentiated with a letter b after name condition (i.e. P b). Tracks for pseudobulk-scATAC-seq are labeled with "sc" after name condition (i.e. P sc). Annotated regulatory elements are indicated: strong enhancer (orange), distal enhancer (yellow) and promoter-TSS (red), Genehancer track (version 2) on the UCSC

Genome Browser(db=hg38). The purple box marks the genomic interval displayed for each gene locus.

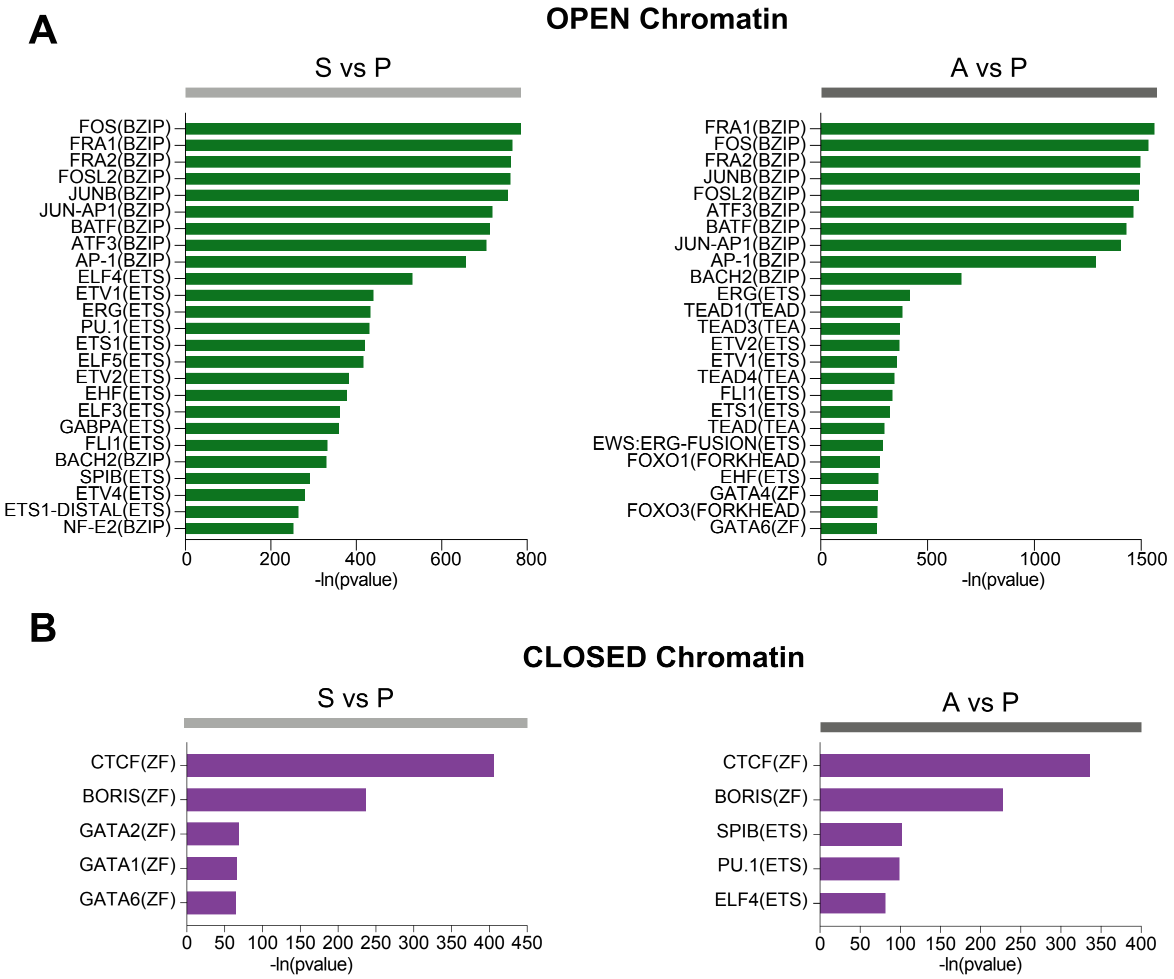
A
OPEN Chromatin
S vs P
FOS(BZIP)
FRA1(BZIP)
FRA2(BZIP)
FOSL2(BZIP)
JUNB(BZIP)
JUN-AP1(BZIP)
BATF(BZIP)
ATF3(BZIP)
AP-1(BZIP)
ELF4(ETS)
ETV1(ETS)
ERG(ETS)
PU.1(ETS)
ETS1(ETS)
ELF5(ETS)
ETV2(ETS)
EHF(ETS)
ELF3(ETS)
GABPA(ETS)
FLI1(ETS)
BACH2(BZIP)
SPIB(ETS)
ETV4(ETS)
ETS1-DISTAL(ETS)
NF-E2(BZIP)
0
200
400
600
800
-ln(pvalue)
A vs P
FRA1(BZIP)
FOS(BZIP)
FRA2(BZIP)
JUNB(BZIP)
FOSL2(BZIP)
ATF3(BZIP)
BATF(BZIP)
JUN-AP1(BZIP)
AP-1(BZIP)
BACH2(BZIP)
ERG(ETS)
TEAD1(TEAD)
TEAD3(TEA)
ETV2(ETS)
ETV1(ETS)
TEAD4(TEA)
FLI1(ETS)
ETS1(ETS)
TEAD(TEA)
EWS:ERG-FUSION(ETS)
FOXO1(FORKHEAD)
EHF(ETS)
GATA4(ZF)
FOXO3(FORKHEAD)
GATA6(ZF)
0
500
1000
1500
-ln(pvalue)
B
CLOSED Chromatin
S vs P
CTCF(ZF)
BORIS(ZF)
GATA2(ZF)
GATA1(ZF)
GATA6(ZF)
0 50 100 150 200 250 300 350 400 450
-ln(pvalue)
A vs P
CTCF(ZF)
BORIS(ZF)
SPIB(ETS)
PU.1(ETS)
ELF4(ETS)
0 50 100 150 200 250 300 350 400
-ln(pvalue)

Supplementary Fig. 6. Chromatin accessibility landscape of iPSC-derived HSPCs.

(A) HOMER motif enrichment analysis of open DAPs. The top 25 enriched transcription factor (TF) binding motifs are shown for DAPs across S (left) and A (right) conditions versus P. Each motif is annotated with the corresponding TF name, TF family and enrichment in *p*-value, as determined by HOMER analysis This highlight condition-specific regulatory dynamics.

(B) HOMER motif enrichment analysis of closed DAPs. The top 5 enriched TF binding motifs are shown for DAPs across S (left) and A (right) conditions versus P. Each motif is annotated with the corresponding TF name, TF family and enrichment in *p*-value, as determined by HOMER analysis This highlight condition-specific regulatory dynamics.

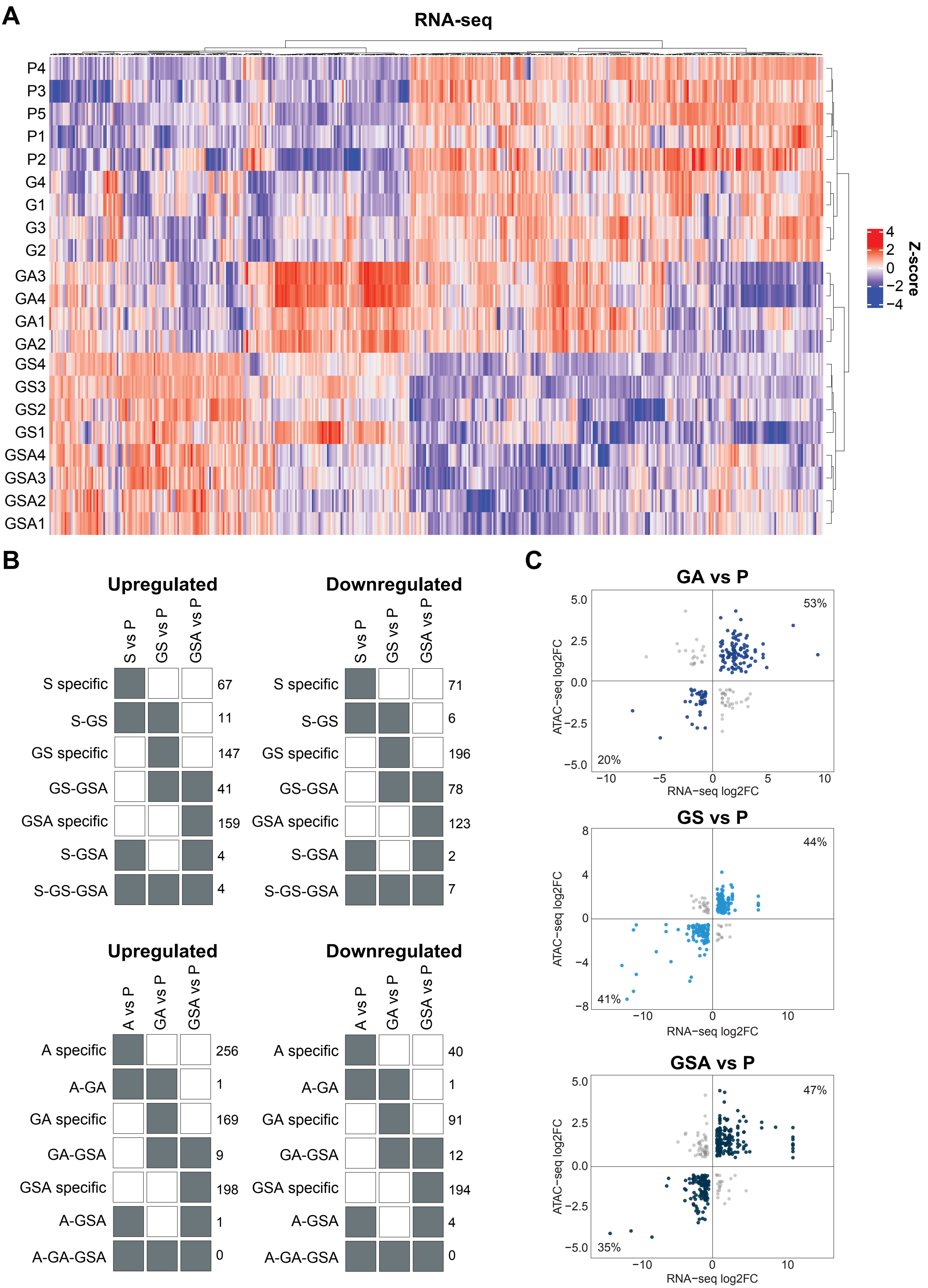
A
RNA-seq
P4
P3
P5
P1
P2
G4
G1
G3
G2
GA3
GA4
GA1
GA2
GS4
GS3
GS2
GS1
GSA4
GSA3
GSA2
GSA1
Z-score
4
2
0
−2
−4
B
Upregulated
S vs P
GS vs P
GSA vs P
S specific 67
S-GS 11
GS specific 147
GS-GSA 41
GSA specific 159
S-GSA 4
S-GS-GSA 4
Downregulated
S vs P
GS vs P
GSA vs P
S specific 71
S-GS 6
GS specific 196
GS-GSA 78
GSA specific 123
S-GSA 2
S-GS-GSA 7
Upregulated
A vs P
GA vs P
GSA vs P
A specific 256
A-GA 1
GA specific 169
GA-GSA 9
GSA specific 198
A-GSA 1
A-GA-GSA 0
Downregulated
A vs P
GA vs P
GSA vs P
A specific 40
A-GA 1
GA specific 91
GA-GSA 12
GSA specific 194
A-GSA 4
A-GA-GSA 0
C
GA vs P
53%
20%
ATAC−seq log2FC
RNA−seq log2FC
GS vs P
44%
41%
ATAC−seq log2FC
RNA−seq log2FC
GSA vs P
47%
35%
ATAC−seq log2FC
RNA−seq log2FC

Supplementary Fig. 7. Differential transcriptomic distribution.

(A) The heatmap depicts the transcriptomic distribution of differentially expressed genes across five experimental conditions: P, G, GA, GS, and GSA. Gene expression levels were normalized and hierarchically clustered to reveal patterns of upregulation and downregulation specific to each condition. Differential gene expression analysis was performed using DESeq2. Significance was assessed with the Wald test (two-sided) on a negative binomial model. P-values were adjusted for multiple comparisons using the Benjamini–Hochberg false discovery rate (FDR).

(B) The intersection representation illustrates the extent of overlap between upregulated and downregulated DEGs in the indicated pairwise comparisons: A vs P, GA vs P, GSA vs P, S vs P, GS vs P, and GSA vs P. Each box represents the distribution of shared DEGs across comparisons, highlighting common and distinct transcriptional responses relative to the P condition. Overlaps were quantified based on consistent directionality (upregulation or downregulation).

(C) Integration of bulk ATAC-seq and RNA-seq. Each dot represents a significant gene with matched RNA-seq (x-axis, log2FC) and ATAC-seq (y-axis, log2FC) values between GA vs P, GS vs P, and GSA vs P conditions. Genes with significant changes detected in both transcriptomic expression and chromatin accessibility are highlighted in navy blue, blue, and dark blue, respectively. Percentages indicate the proportion of genes in the corresponding quadrants. Grey points indicate genes without concordant or significant changes in both datasets.

P=Parental, S=SETBP1 mutant, A=ASXL1 mutant, G=GATA2 mutant, GS= GATA2-SETBP1 mutant, GA= GATA2-ASXL1 mutant, GSA = GATA2-SETBP1-ASXL1 mutant.
