# Supplementary material for: Human iPSCs-based modeling unveils SETBP1 as a driver of chromatin rewiring in GATA2 deficiency
Source: Nat Commun. 2025 Nov 17;16:10035. doi: 10.1038/s41467-025-65806-9 (PMC12623428; doi:10.1038/s41467-025-65806-9)
Supplement: Supplementary file 2 — Description of Additional Supplementary Files [file 41467_2025_65806_MOESM2_ESM.pdf]

Description of Additional Supplementary Files

File name: Supplementary Data 1

Description: DAPs bulk ATAC-seq

Contains differential accessible peaks (DAPs) from bulk ATAC-seq comparisons between different experimental groups (G, GA, GS, GSA, A, S vs P). Includes gene name, genomic coordinates, log2 fold change, adjusted p-values, and peak annotation.

File name: Supplementary Data 2

Description: DAPs single-cell ATACseq

Lists differential accessible peaks identified from single-cell ATAC-seq analyses (G, GA, GS, GSA vs P). Provides gene-level information including log2FC, p-values, genomic regions, and functional annotations.

File name: Supplementary Data 3

Description: DAPs pseudobulk ATAC-seq

Presents differential accessible peaks derived from pseudobulk analysis of single-cell ATACseq, integrated with bulk ATAC-seq. Contains gene names, log2 fold change, adjusted pvalues, genomic coordinates, and annotations.

File name: Supplementary Data 4

Description: 229 GATA2 target regions

Catalog of genomic regions identified as GATA2 targets by intersecting ATAC-seq data with published ChIP-seq (Fujiwara et al., 2009). Lists genomic coordinates and associated gene names.

File name: Supplementary Data 5

Description: 251 GATA2 target regions (FIMO analysis)

Regions enriched for GATA motifs identified using FIMO motif analysis. Provides chromosome coordinates, associated genes, and number of GATA2 motifs per region.

File name: Supplementary Data 6

Description: DEGs bulk RNA-seq

Differentially expressed genes (DEGs) from bulk RNA-seq across comparisons (G, GA, GS, GSA, A, S vs P). Includes base mean expression, log2 fold change, p-values, adjusted p-values, and statistics.

File name: Supplementary Data 7

Description: Single-cell barcodes and oligos

Contains oligonucleotide sequences and barcodes used for SHARE-seq single-cell experiments. Includes linker sequences, synthesis details, purification method, and barcode assignments across multiple plates.
