# Supplementary material for: Human iPSCs-based modeling unveils SETBP1 as a driver of chromatin rewiring in GATA2 deficiency
Source: Nat Commun. 2025 Nov 17;16:10035. doi: 10.1038/s41467-025-65806-9 (PMC12623428; doi:10.1038/s41467-025-65806-9)
Supplement: Supplementary file 10 — Reporting Summary [file 41467_2025_65806_MOESM10_ESM.pdf]

nature portfolio

Corresponding author(s): Alessandra Giorgetti/Oskar Marin-Bejar

Last updated by author(s): Oct 2, 2025

Reporting Summary

Nature Portfolio wishes to improve the reproducibility of the work that we publish. This form provides structure for consistency and transparency in reporting. For further information on Nature Portfolio policies, see our Editorial Policies and the Editorial Policy Checklist.

Statistics

For all statistical analyses, confirm that the following items are present in the figure legend, table legend, main text, or Methods section.

n/a	Confirmed	
☐	☒	The exact sample size (*n*) for each experimental group/condition, given as a discrete number and unit of measurement
☒	☐	A statement on whether measurements were taken from distinct samples or whether the same sample was measured repeatedly
☐	☒	The statistical test(s) used AND whether they are one- or two-sided<br>*Only common tests should be described solely by name; describe more complex techniques in the Methods section.*
☒	☐	A description of all covariates tested
☒	☐	A description of any assumptions or corrections, such as tests of normality and adjustment for multiple comparisons
☐	☒	A full description of the statistical parameters including central tendency (e.g. means) or other basic estimates (e.g. regression coefficient) AND variation (e.g. standard deviation) or associated estimates of uncertainty (e.g. confidence intervals)
☐	☒	For null hypothesis testing, the test statistic (e.g. *F*, *t*, *r*) with confidence intervals, effect sizes, degrees of freedom and *P* value noted<br>*Give P values as exact values whenever suitable.*
☒	☐	For Bayesian analysis, information on the choice of priors and Markov chain Monte Carlo settings
☒	☐	For hierarchical and complex designs, identification of the appropriate level for tests and full reporting of outcomes
☐	☒	Estimates of effect sizes (e.g. Cohen's *d*, Pearson's *r*), indicating how they were calculated

Our web collection on statistics for biologists contains articles on many of the points above.

Software and code

Policy information about availability of computer code

Data collection

Sortings for ATAC-seq, scATAC-seq and RNA-seq were performed on MoFlo Astrios EQ, Cell sorter Beckman Coulter instrument

Data analysis

Regarding ATAC-seq analysis:
nf-core/atacseq – v2.1.2
Trim-Galore
BWA (Burrows-Wheeler Alignment tool)
SAMtools
BEDTools
bedGraphToBigWig
MACS2
HOMER
featureCounts
MultiQC
FastQC
R – v4.2.0
edgeR – v3.38.1
limma – v3.52.1
ComplexHeatmap – v2.12.1
deepTools – v3.5.6

Regarding scATAC-seq:
FastQC – v0.12.1
Cutadapt – v4.9
Bowtie2 – v2.5.4
SAMtools – v1.14
BEDTools – v2.30.0
deepTools – v3.5.5
R – v4.4.2
Signac – v1.14.0
Seurat – v5.2.1
MACS2 – v2.2.9.1
sva (ComBat_seq) – v3.44.0
DESeq2 – v1.36.0

Regarding RNA-seq:
nf-core/rnaseq – v3.10.1
FastQC – (v0.12.1)
MultiQC
STAR
RSEM
R – v4.2.0
DESeq2 – v1.36.0
sva (ComBat_seq) – v3.44.0

For manuscripts utilizing custom algorithms or software that are central to the research but not yet described in published literature, software must be made available to editors and reviewers. We strongly encourage code deposition in a community repository (e.g. GitHub). See the Nature Portfolio guidelines for submitting code & software for further information.

Data

Policy information about availability of data

All manuscripts must include a data availability statement. This statement should provide the following information, where applicable:

- Accession codes, unique identifiers, or web links for publicly available datasets
- A description of any restrictions on data availability
- For clinical datasets or third party data, please ensure that the statement adheres to our policy

The accession numbers for the RNA-seq, ATAC-seq, and scATAC-seq data reported in this paper are GEO (Gene Expression Omnibus): GSE265824, GEO: GSE264140, and GEO: GSE300314, respectively.

Research involving human participants, their data, or biological material

Policy information about studies with human participants or human data. See also policy information about sex, gender (identity/presentation), and sexual orientation and race, ethnicity and racism.

Reporting on sex and gender	n/a
Reporting on race, ethnicity, or other socially relevant groupings	n/a
Population characteristics	n/a
Recruitment	n/a
Ethics oversight	n/a

Note that full information on the approval of the study protocol must also be provided in the manuscript.

Field-specific reporting

Please select the one below that is the best fit for your research. If you are not sure, read the appropriate sections before making your selection.

[x] Life sciences [ ] Behavioural & social sciences [ ] Ecological, evolutionary & environmental sciences

For a reference copy of the document with all sections, see nature.com/documents/nr-reporting-summary-flat.pdf

Life sciences study design

All studies must disclose on these points even when the disclosure is negative.

Sample size	No sample size calculation was used, however each experiment has the minimum samples to calculate normalization and apply the necessary statistical tests
Data exclusions	No data were excluded from this manuscript
Replication	Each reported experiment was performed at least in three independent experiments. All replications were successful.
Randomization	Allocation into experimental groups was not applicable to this study. We worked with established cell lines carrying known genetic alterations, and grouping was defined based on their mutational status rather than random allocation. Since the identity and genotype of each cell line were predetermined, randomization or covariate control was not relevant.
Blinding	We did not perform any blind experiment as each line has different mutations from the others and they were genotyped to keep control that there were not cross line contamination.

Reporting for specific materials, systems and methods

We require information from authors about some types of materials, experimental systems and methods used in many studies. Here, indicate whether each material, system or method listed is relevant to your study. If you are not sure if a list item applies to your research, read the appropriate section before selecting a response.

Materials & experimental systems

n/a	Involved in the study	
☐	☒	Antibodies
☐	☒	Eukaryotic cell lines
☒	☐	Palaeontology and archaeology
☒	☐	Animals and other organisms
☒	☐	Clinical data
☒	☐	Dual use research of concern
☒	☐	Plants

Methods

n/a	Involved in the study	
☒	☐	ChIP-seq
☐	☒	Flow cytometry
☒	☐	MRI-based neuroimaging

Antibodies

Antibodies used	For Flow cytometry, following antibodies were used: CD34-PE (Miltenyi Biotec S.L., clone 8G12, #130046702), CD43-APC (Becton Dickinson, clone 1G10, #560198), CD45-APC-H7 (Becton Dickinson, clone 2D1, #560178), CD33-APC (Becton Dickinson, clone WM53, #551378), CD11b-PE-Cy7 (Becton Dickinson, clone ICRF44, #557743), CD14-BV421 (Becton Dickinson, clone MPHIP9, #563743) and CD15-PE (Becton Dickinson, clone HI98, #555402). Dead cell exclusion was achieved using 7-Aminoactinomycin D stain (7AAD) (Invitrogen, #92008). Annexin V-FITC was used to measure apoptosis (Immunostep, #ANXVF-200T).<br>For Fluorescence activated cell sorting (FACS), following antibodies were used: CD34-FITC (Becton Dickinson, clone 8G12, #348053), CD43-APC, (Becton Dickinson, clone 1G10, #560198), CD45-APC-H7 (Becton Dickinson, clone 2D1, #560178), CD33-BV510 (Becton Dickinson, clone WM53, #563257). Dead cell exclusion was achieved using Propidium iodide (Sigma, #P4170-100MG).
Validation	Established antibodies were validated in house by FACS with single staining. In immunostainings antibodies were tested by comparing the signal to secondary only control, or by comparing the expression pattern with published data sets.

Eukaryotic cell lines

Policy information about cell lines and Sex and Gender in Research

Cell line source(s)	ESi086-A iPSC cell line were used for generation of mutant iPSC. ESi806-A-1 were also used as is the GATA p.R396Q mutant cell line. All lines carry 46, XY karyotype.
Authentication	Karyotyping - The genomic integrity of the iPSC line was evaluated by G-banded metaphase analysis (300–500 bands) at Sant Joan de Deu, Barcelona. 70% confluent hiPSC colonies were incubated with KaryoMax colcemid (Invitrogen, #15212012), trypsinized, treated with hypotonic solution and fixed in 75% methanol + 25% acetic acid. A minimum of 20 metaphases were examined.<br><br>Pluripotency markers expression . iPSC were fixed with 4% PFA for 20 at RT, blocked and permeabilized with TBS + 0.5% Triton X-100 + 6% donkey serum at 1h at RT. Primary antibodies were incubated overnight in TBS + 0.1% Triton X-100 + 6% donkey serum at 4 °C, and secondary antibodies were incubated 2 h at 37 °C. Nuclei were stained using 4,6-diamino-2-fenilindol (DAPI).

Mycoplasma contamination	All cell lines tested negative in mycoplasma tests.
Commonly misidentified lines (See ICLAC register)	n/a

Plants

Seed stocks	n/a
Novel plant genotypes	n/a
Authentication	n/a

Flow Cytometry

Plots

Confirm that:

- [x] The axis labels state the marker and fluorochrome used (e.g. CD4-FITC).
- [x] The axis scales are clearly visible. Include numbers along axes only for bottom left plot of group (a 'group' is an analysis of identical markers).
- [x] All plots are contour plots with outliers or pseudocolor plots.
- [x] A numerical value for number of cells or percentage (with statistics) is provided.

Methodology

Sample preparation	EBs were dissociated as single cell using Cell Dissociation Buffer (Life Technologies S.A., #13151014). Cells were spun at 300 x g for 5 minutes and washed once with FACS buffer (PBS + 0.5 mM EDTA + 2% HSA (Vitrolife, #10064)). For surface staining, cells were incubated with fluorochrome-conjugated monoclonal antibodies specific to the markers for 15 min at RT in the dark. Following incubation, cells were washed twice with FACS buffer and resuspended. After flow cytometry sample preparation cells were resuspended in Annexin V buffer (Immunostep, ANXVF-200T) following manufacturer's protocol. Gallios Flow Cytometer (Beckman Coulter) was used to run samples. The positive population was gated using Kaluza Analysis Software (Beckman Coulter).
Instrument	Flow cytometry Analyzer: Gallios Beckman Coulter Analyzer<br>FACS sorter: MoFlo Astrios EQ, Cell sorter Beckman Coulter instrument
Software	Flow Cytometry/FACS plots were generated using Kaluza Analysis 2.4.0.20305
Cell population abundance	FACS sorters were used in the "purity" setting.
Gating strategy	First, using FSC-A and SSC-A cell debris were excluded. Then SSC-A and Viability die (7AAD/PI) were used to keep alive cells. Then, using SSC-H and SSC-A we gated single cells. In the first experiement, single color controls were used to establish cut off lines for each gate.

- [x] Tick this box to confirm that a figure exemplifying the gating strategy is provided in the Supplementary Information.
